# Bringing theory to life: integrating case-based learning in applied physiology for undergraduate physiotherapy education

**DOI:** 10.1186/s12909-025-06725-7

**Published:** 2025-02-13

**Authors:** Rehab E. Abo Elgheit, Nagwa Nashat

**Affiliations:** 1 Departments of Physiology, Faculty of Medicine, Tanta University, El Geesh Street, Tanta, Egypt; 2 Departments of Physiology, Faculty of Physical Therapy, AlSalam University, Tanta, Egypt; 3 Department of Family Medicine, Faculty of Medicine, Menoufia University, Menoufia, Egypt; 4 Quality Assurance Center, Menoufia National University, Menoufia, Egypt

**Keywords:** Case-based learning, Applied physiology, Physiotherapy, Perception, Academic achievements

## Abstract

**Background:**

Case-based learning (CBL) is considered an effective teaching approach that provides medical students with a learning environment simulating future actual practice. So we aimed to implement CBL, as an additional component to traditional teaching methods (TTMs) in applied physiology for undergraduate physiotherapy students (UPS) and to evaluate its impact on students’ perception and academic performance compared to TTMs alone.

**Methods:**

CBL was implemented during the teaching of applied physiology throughout the neuroscience course in the 4th semester for UPS. Nine cases related to the topics taught were selected. The academic achievement was evaluated through the students’ grades, and the students’ perception and facilitators’ feedback were explored through a structured, web-based perception questionnaire.

**Results:**

Of the 244 UPS students, 238 completed the survey following the CBL approach. A total of 97.5% reported a higher perception of the combined CBL with TTMs compared to the traditional approach, with a median overall perception score of 99.0. 93.3% of the enrolled students ensured that combined CBL with TTMs was better in all features in applied physiology education. Integrating CBL with TTMs was associated with improved academic performance. While the maximum grade remained consistent at 10 for both traditional physiology labs and combined CBL with TTMs, the minimum grade improved significantly from 2.0 in traditional lab teaching to 7.5 in combined CBL and TTMs. Median grades also increased from 8.5 to 10.0 when CBL was combined with TTMs, with better performance observed at both the 25th and 75th percentiles under the hybrid CBL and TTMs approach (10.0 vs. 7.0 and 10.0 vs. 9.5, respectively).

**Conclusion:**

Incorporating CBL alongside TTMs provided a more engaging learning experience, with increased students’ perception, and promoted their academic achievement. An enhanced teaching framework with the integration of CBL can be broadly implemented as a more interactive teaching tool not only in applied physiology but also in other health sciences to overcome the limitations of the TTMs and ensure better outcomes.

**Supplementary Information:**

The online version contains supplementary material available at 10.1186/s12909-025-06725-7.

## Introduction

The fundamental medical sciences, including applied physiology, constitute a foundational element of medical curricula, providing essential understanding of the human body’s biological underpinnings, various diseases, and associated therapies [1].

Physiotherapy students rely on this basic knowledge as they develop their clinical expertise. However, there is growing concern that TTMs in physiotherapy education fail to yield optimal learning outcomes [2]. Integrating basic sciences with clinical relevance from the outset of education is believed to enhance information retention and facilitate its application in clinical settings [1].

In the traditional framework of physiotherapy education, basic sciences, including medical physiology, are typically taught in the initial two years of undergraduate studies with limited interdisciplinary interaction. This approach may detrimentally impact students’ perception and long-term retention of foundational scientific knowledge [3].

The inability to connect foundational knowledge with clinical contexts may result in graduates lacking critical thinking and problem-solving skills essential for effective clinical practice [4]. Moreover, senior undergraduate physiotherapy students frequently express informal dissatisfaction with their memory of basic medical sciences and struggle to correlate this content with later clinical curricula [5].

As physiotherapy students’ progress through their education, their perceptions of foundational courses often become increasingly negative, highlighting a potential flaw in the educational system where acquired knowledge risks becoming inaccessible and inert [6]. Research indicates that basic science knowledge acquired within a clinical context is more readily applied and comprehended by students [1].

Despite significant efforts over decades, the practical implementation of integration remains challenging. Case-based learning (CBL) emerges as a promising alternative, characterized by interactivity and student autonomy, potentially fostering greater enthusiasm for learning [7–9].

Recent studies have explored the potential benefits of combining CBL with TTMs in physiotherapy education, contrasting with traditional didactic lectures and practical classes that are often teacher-centered with minimal student engagement [10, 11].

Monitoring student perception throughout undergraduate courses may inform recommendations for better integration of basic sciences within clinical subjects, facilitating the unified application of foundational knowledge to clinical scenarios. By integrating CBL approach alongside TTMs in applied physiology, for physiotherapy students may enhance students’ ability to apply theoretical knowledge to clinical scenarios, thereby improving their academic performance and perception.

Hence, the current study aims to integrate CBL with TTMs in teaching applied physiology for undergraduate physiotherapy students and evaluate the impact of this combined hybrid approach on student perceptions and academic performance, comparing it to the application of TTMs alone.

## Material and methods

### Study design

This is an interventional study that was conducted at the Faculty of Physiotherapy, AlSalam University, during the period of January to May 2023, on the undergraduate physiotherapy students during the neuroscience course.

### Ethical considerations

This study was conducted in accordance with the Declaration of Helsinki.

### Setting and participants

#### Study participants and eligibility

A cohort comprising 244 undergraduate physiotherapy students in their fourth semester, who were enrolled in a neuroscience course were recruited for the present study. There were no exclusion criteria.

### Sample size

The study included all fourth-semester students (*n* = 244), with an expected dropout rate of 5%. A formal sample size calculation was not performed, as the objective was to include all eligible students.

### The facilitators

Nine volunteer physiologists with expertise in applied physiology served as facilitators for the CBL sessions. Most had prior experience with interactive teaching methodologies, including problem-based learning (PBL) and small-group discussions.

#### Facilitators training

To ensure effective CBL implementation, the facilitators underwent a comprehensive orientation and training program selectively tailored for applied physiology teaching through CBL. The training program spanned over two weeks and covered the following key dimensions:

##### Theoretical training and orientation sessions

Included an in-depth understanding of the CBL methodology. The training focused on clarification of the CBL principles in applied physiology. They received guidance on their roles and responsibilities through CBL sessions, including facilitating knowledge delivery through case scenarios and discussions. They were encouraged to act as guides rather than teachers. The facilitators were trained to actively monitor group dynamics, check their progress, supervise their discussions, encourage the active participation of students, and provide guidance as needed.

Facilitators were trained for the critical analysis of clinical cases, formulating relevant questions that trigger critical thinking, and how to guide discussions to maximize students’ learning.

##### Practical training

Hands-on workshops simulating CBL sessions were arranged, where facilitators practiced moderating discussions, monitoring group dynamics, and guiding students through case analyses. Additionally, facilitators have received training in formulating relevant questions that trigger critical thinking and guide discussions.

##### Assessment of preparedness

Facilitators participated in mock sessions, where their skills in engaging students, encouraging participation, and managing discussions were evaluated and refined.

##### Ongoing professional support for facilitators

Throughout the semester, facilitators benefited from a comprehensive support system that included regular mentoring sessions and weekly debriefing meetings to address emerging challenges. A robust peer support network was established among facilitators, fostering collaborative problem-solving and shared learning experiences. Additionally, facilitators had continuous access to curated educational resources and materials to enhance their teaching effectiveness and maintain high-quality CBL implementation.

##### Facilitator selection process

All facilitators were selected from the department of physiology faculty members who met the following criteria:

- ▪ Expertise in applied physiology: Minimum of 5 years of teaching experience in applied physiology
- ▪ Prior experience with interactive teaching methods including PBL or case-based teaching methods
- ▪ Active involvement in physiotherapy education
- ▪ Demonstrated interest in innovative teaching methodologies

##### Expert-led comprehensive facilitator training program

The intensive CBL facilitator training program was conducted by multidisciplinary education experts as follow:

- ▪ A senior professor with more than fifteen years of CBL implementation experience
- ▪ An educational expert specializing in medical education methodologies
- ▪ A curriculum development specialist

### Effective CBL session

#### Aligning goals: building the learning objectives of effective CBL session

The learning objectives for each CBL session were collaboratively developed by a committee of physiologists and neurologists, together with the facilitators, based on the overall course objectives and the intended outcomes (ILOs) of the neuroscience module. To ensure that students gained both theoretical knowledge and practical skills, ILOs of each CBL session were aligned with the course learning outcomes (CLOs), including knowledge and skill-based objectives related to neurophysiology. This alignment ensured that the cases addressed both theoretical understanding and practical application to clinical scenarios.

These objectives were clearly defined to the facilitators after sharing the case study scenario and all relevant patient information in each CBL. The learning objectives were shared with students at the beginning of each CBL session to guide discussions and ensure alignment with the session’s goals. During the discussions, students were encouraged to ask questions and explore related topics, fostering an environment of active learning and critical thinking [12].

#### CBL as an integral part of physiology course

CBL sessions were integrated as a mandatory component of the applied physiology course in the 4^th^ semester of the undergraduate physiotherapy program. This course is part of the foundational phase of the program, which spans the first four semesters and focuses on basic and applied sciences. The entire undergraduate physiotherapy program consists of 10 semesters over five academic years, with an additional internship year comprising 36 h per week for 12 months.

An overview of case-related physiology topics was delivered to students in their traditional interactive dictated lectures. These traditional lectures were used to deliver foundational knowledge in applied physiology. The CBL sessions were then implemented during the laboratory component of the course, allowing students to apply theoretical concepts from lectures to clinical case scenarios.

These CBL sessions were conducted weekly and designed to provide hands-on, case-based problem-solving experiences aligned with the topics covered in the neuroscience course. Furthermore, the evaluation of students’ understanding of applied physiology, including their participation in CBL sessions, is carried out through Objective Structured Practical Examinations (OSPE).

The interactive didactic lectures and practical labs were retained in the 4^th^ semester and were consistent with TTMs in the first semester. The frequency of interactive lectures was not reduced; instead, CBL was integrated into the weekly lab sessions, which were already part of the curriculum.

#### CBL formatting

Nine cases were selected through online search to match the specific objectives and contents of the neuroscience physiology course. Each case was thoroughly reviewed, focusing on relevance, comprehensiveness, and the potential to stimulate critical thinking and discussion among students. ILOs of each CBL case were explicitly designed to address CLOs, including knowledge and skill-based objectives (Table 1).

**Table 1:** The selected cases for implementing case-based learning in applied physiology

No of case	Case title	Relevant course objectives	Reference
**1.**	**Brown-Séquard syndrome (BSS) with Horner’s syndrome (HS)**	➲ Relate the manifestations of Horner’s syndrome with autonomic dysfunction ➲ Demonstrate the impact of spinal cord lesions on sensory, motor, and autonomic functions, particularly in the context of BSS	[13]
**2.**	**Diabetic polyneuropathy (DN)**	➲ Correlate the metabolic disturbance in diabetic patients with polyneuropathy ➲ Identify the key criteria for pain in DN ➲ Describe the mechanisms of pain modulation in DN	[14]
**3.**	**Thalamic Infarction with Cheiro-Oral Syndrome**	➲ Explain the physiological basis of the sensory deficits associated with thalamic syndrome ➲ Organize the sensory and motor functions of the thalamus ➲ Relate thalamic lesions to various sensory deficits	[15]
**4.**	**Referred pain**	➲ Explain the physiology of referred pain in acute appendicitis ➲ State the mechanisms of referred pain ➲ Correlate the origin of the pain with the referred skin dermatome	[16]
**5.**	**Pain Analgesia**	➲ Distinguish the different levels of the endogenous pain analgesia system ➲ Analyze the physiological mechanisms through which acupuncture could provide therapeutic benefit in pain relief	[17]
**6.**	**Post COVID-19 Positive Babinskis sign**	➲ Explain the physiological basis of the positive Babinski sign in neurological diseases ➲ Link the impact of various infections on the pyramidal tract with the positive Babinski sign	[18]
**7.**	**Ankle clonus (AC)**	➲ Discuss the physiological base of clonus ➲ Delineate the clinical significance of AC and its diagnostic value ➲ Assess the potential role of various physiotherapy modalities in facilitating motor recovery in clonus	[19]
**8.**	**Hung-up knee jerk**	➲ Interpret the findings of deep tendon reflexes in clinical settings ➲ Discuss the diagnostic value of the abnormal knee jerk	[20]
**9.**	**Post-Covid Facial Palsy**	➲ Explain the potential impact of various infections on facial nerve functions ➲ Identify the manifestations of facial palsy	[21]

#### The students’ preparation for CBL sessions

To ensure student preparedness, all cases- related materials and recommended reading resources were made available on Microsoft Teams at the beginning of the semester. Students were expected to review these materials, engage in pre-reading, and come to the session ready for active participation and meaningful discussion. This preparatory phase was vital for fostering effective group analysis and ensuring all students had a foundational understanding of the case content.

#### Optimizing CBL in physiology labs: small groups’ organization and facilitators’ guidance

In the physiology labs, the total number of students was organized into small groups, each comprising 25 to 30 students. These groups were further divided into approximately five smaller batches, each with six students. This setup allowed for increased interaction and personalized attention.

Nine trained physiologists facilitated the CBL sessions, rotating among the different lab groups to provide students with a variety of expert insights and to ensure consistent guidance and support during the CBL activities. Each facilitator remained with a small group for the entire duration of the lab session, actively supervising and moderating discussions. By rotating across various lab groups at different time slots throughout the week, facilitators ensured that all students received consistent, high-quality guidance. This rotational approach effectively accommodated the large number of participants while preserving the small-group dynamic critical for the success of CBL implementation.

#### CBL implementation: the Kaddoura approach in action

The facilitator guided the learning process during the CBL sessions using the Kaddoura approach (Supplementary Figure 1). The Kaddoura method includes five sequential steps: case presentation, presentation of triggering questions by the facilitators, creating a comfortable and safe atmosphere for learners, active participation of all students in discussions, and finally case summarization by the facilitator. Each session started with an interactive introduction to the physiology topic. All students were encouraged to participate actively in the discussions [22].

### Tools of data collection: the data was collected through the following tools

#### The students’ perception questionnaire

To better understand the students’ acceptance and perception of the newly implemented CBL approach, they were encouraged to complete a web-based questionnaire using Google Forms. It was delivered at the end of the fourth semester to physiotherapy students who enrolled in the neuroscience course. The questionnaire was administered in English. The questionnaire consisted of two sections: the first section included demographic information, while the second section was specially designed to evaluate students’ perception with the CBL approach, as follow:

##### Socio-demographic (SD) section of questionnaire

A SD part was prepared to ask about the participants’ gender and age.

##### The perception (P) section of questionnaire

This part was composed of 20 items and aimed to gather insights on perception levels and experiences with combined CBL and TTMs, that was conducted during the fourth semester, using a five-point Likert scale, including (strongly agree, agree, neither agree nor disagree, disagree, and strongly disagree).

The face validity index (FVI) of P questionnaires was evaluated to assess both clarity and comprehensibility of the P questionnaires items, achieving an overall score of 0.95. This was performed by 20 users. The students were asked to rate each item by score from 1 (not clear- not understandable item) to 4 (understandable and clear item). Scores of (3 and 4) were categorized as 1 (means clear and understandable) and scores of 1 and 2 were categorized as 0 (understandable or not clear). The FVI for each item was then computed, and the average was taken to obtain the scale’s average FVI score [23]. Moreover, the content validity index (CVI) and content validity ratio (CVR) of the P questionnaires were estimated at 0.81 and 0.80, respectively. They were confirmed as being more than 0.60. Cronbach’s alpha of the P questionnaire was (0.9) [22]. Finally, a question was included to rate the overall perception level of the CBL approach on a scale of (0 to 10) [22].

The students were asked to rate each instruction approach on a five-item Likert-type scale regarding the compatibility of each statement with combined CBL and TTMs approach. A total score for each participant was obtained from the P questionnaire and divided by (20) in order to calculate a mean perception score over 5 to be applied in statistical analysis [24].

#### Facilitators’ feedback questionnaire

The Facilitators’ feedback questionnaire had both open-ended and structured questions. The open-ended questions were developed to help in the better CBL implementation in the future through collection of facilitators’ insights on challenges and recommendations for improving CBL implementation. While the close-ended questions were based on nine parameters and scored using a five-point Likert scale ranging from strongly disagreeing to strongly agreeing [25]. The facilitators’ feedback questionnaire was validated through a pilot test with a small group of facilitators and experts in the field, achieving FVI score of 0.92, indicating high face validity. Additionally, the CVI and CVR were estimated at 0.85 and 0.79, respectively, indicating good content validity of the feedback questionnaire.

#### Indicators for academic achievement

To compare academic performance between TTMs alone and the combined case-based and traditional education in applied physiology, examination scores obtained by physiotherapy students at the end of the first and fourth semesters were used as benchmarks.

### Statistical methods

All data were tabulated and analyzed by the statistical package for the social sciences software program, IBM SPSS Statistics for Windows, version 26 (IBM Corp., Armonk, N.Y., USA). The total perception score was calculated by giving 5 to strongly agree, 4 to agree, 3 to neutral, 2 to disagree, and 1 to strongly disagree responses. A perception score of 3 or above on the 5-point Likert scale was considered positive, while scores below 3 were considered negative. Categorical data were represented as frequencies and percentages. Possible associations between categorical variables were analyzed using Pearson’s Chi-Square test or Fisher’s exact tests as appropriate. Continuous variables were reported as median with interquartile range (IQR: 25^th^ – 75^th^ percentiles) and were compared using the Mann–Whitney U test because they were not normally distributed. Furthermore, Wilcoxon-signed-rank test was applied to compare the student’s grades before and after CBL method intervention. Open-ended responses from the facilitators’ feedback questionnaire were analyzed using thematic analysis. Data familiarization was followed by coding to identify recurring patterns and unique insights. Thematic analysis was conducted to group related codes into broader themes, which were categorized under relevant domains. A *p-*value of < *0.05* was considered statistically significant.

## Results

### Demographic data

Two hundred thirty-eight out of 244 undergraduate physiotherapy students completed the survey following the combined CBL and TTMs approach during the neuroscience course in the fourth semester. 72.3% of participants were female, compared to 27.7% male. Of whom, 90.80% were 18–20 years old, while 9.2% were 21–23 years old (Figure S2).

### Student’s perception level with the different features of hybrid learning “ combined CBL with TTMs” (Table 2)

**Table 2:** Student opinions regarding the different features of the hybrid approach integrating CBL with traditional teaching methods in comparison to traditional learning (*N* = 238)

CBL features	Agree and strongly agree		Neutral		Disagree and strongly disagree	
	**N**	**%**	**N**	**%**	**N**	**%**
**1. Covers objectives well**	234	98.3%	1	0.4%	3	1.3%
**2. It is more interesting**	231	97.1%	4	1.7%	3	1.3%
**3. Increases students’ comprehension**	228	95.8%	7	2.9%	3	1.3%
**4. More cooperation and participation from students**	223	93.7%	10	4.2%	5	2.1%
**5. It is closer to reality**	232	97.5%	4	1.7%	2	0.8%
**6. Increases students’ motivation to learn**	231	97.1%	6	2.5%	1	0.4%
**7. Facilitates students’ learning**	229	96.2%	6	2.5%	3	1.3%
**8. Information is well organized**	228	95.8%	6	2.5%	4	1.7%
**9. It is more practical**	222	93.3%	11	4.6%	5	2.1%
**10. The learned material has higher persistence**	229	96.2%	7	2.9%	2	0.8%
**11. Increases students’ visualization power**	227	95.4%	8	3.4%	3	1.3%
**12. Most nursing courses can be offered through CBL method**	233	97.9%	4	1.7%	1	0.4%
**13- Increases students’ self-confidence**	230	96.6%	7	2.9%	1	0.4%
**14. Reduces class monotony**	230	96.6%	6	2.5%	2	0.8%
**15. Makes students think deeply**	229	96.2%	7	2.9%	2	0.8%
**16. There is possibility for more questions and answers**	233	97.9%	4	1.7%	1	0.4%
**17. If this method is used in clinical practice, it will be more efficient**	231	97.1%	4	1.7%	3	1.3%
**18. Students’ information is well assessed and evaluated**	234	98.3%	3	1.3%	1	0.4%
**19. Summarizing the contents is easy**	231	97.1%	5	2.1%	2	0.8%
**20. In general, CBL method is better than lecture-based learning**	232	97.5%	4	1.7%	2	0.8%

*CBL* case-based learning, *N* Number

From the perspective of the enrolled students, the most significant advantages of the integrating CBL with TTMs as a hybrid teaching tool, compared to TTMs alone, were its ability to comprehensively cover course objectives and effectively evaluate students’ knowledge (98.3%). Additionally, most participants (97.9%) believed that the CBL approach allowed for greater engagement through more questions, and 97.5% stated that CBL felt closer to real-life scenarios. A majority of students (97.1%) described CBL as a motivating, efficient, and engaging teaching tool in clinical practice that effectively summarizes content.

Additionally, 96.6% of participants stated that CBL enhances self-confidence and reduces class monotony. Among the 238 undergraduate students, 229 highlighted that CBL significantly facilitates learning, promotes deep thinking, and improves retention of topics. Furthermore, 95.8% noted that CBL organizes information well and supports comprehension, while 95.4% believed it enhances visualization skills. Cooperation and participation were also cited as benefits by 93.7%, and 93.3% ensured that CBL is a more practical tool.

### Association between gender and perception level regarding individual features of incorporating CBL to the traditional learning methods (Table 3)

**Table 3:** The association between gender and perception level about various features of the case-based learning

		**Gender**				**Fisher Exact test**	
		**Female**		**Male**		**X**^**2**^	***P-Value***
**1. Covers objectives well**	**A and SA**	169	98.3%	65	98.5%	0.758	1.00
	**N**	1	0.6%	0	0.0%		
	**D and SD**	2	1.2%	1	1.5%		
**2. It is more interesting**	**A and SA**	169	98.3%	62	93.9%	4.366	0.085
	**N**	1	0.6%	3	4.5%		
	**D and SD**	2	1.2%	1	1.5%		
**3. Increases students’ comprehension**	**A and SA**	166	96.5%	62	93.9%	1.336	0.513
	**N**	4	2.3%	3	4.5%		
	**D and SD**	2	1.2%	1	1.5%		
**4. More cooperation and participation from students**	**A and SA**	163	94.8%	60	90.9%	2.644	0.286
	**N**	5	2.9%	5	7.6%		
	**D and SD**	4	2.3%	1	1.5%		
**5. It is closer to reality**	**A and SA**	168	97.7%	64	97.0%	1.604	0.471
	**Neutral**	2	1.2%	2	3.0%		
	**D and SD**	2	1.2%	0	0.0%		
**6. Increases students’ motivation to learn**	**A and SA**	168	97.7%	63	95.5%	2.064	0.532
	**N**	3	1.7%	3	4.5%		
	**D and SD**	1	0.6%	0	0.0%		
**7. Facilitates students’ learning**	**A and SA**	164	95.3%	65	98.5%	2.276	0.341
	**N**	6	3.5%	0	0.0%		
	**D and SD**	2	1.2%	1	1.5%		
**8. Information is well organized**	**A and SA**	165	95.9%	63	95.5%	1.436	0.552
	**N**	5	2.9%	1	1.5%		
	**D and SD**	2	1.2%	2	3.0%		
**9. It is more practical**	**A and SA**	160	93.0%	62	93.9%	1.998	0.348
	**N**	7	4.1%	4	6.1%		
	**D and SD**	5	2.9%	0	0.0%		
**10. The learned material has higher persistence**	**A and SA**	165	95.9%	64	97.0%	0.498	1.000
	**N**	5	2.9%	2	3.0%		
	**D and SD**	2	1.2%	0	0.0%		
**11. Increases students’ visualization power**	**A and SA**	164	95.3%	63	95.5%	1.203	0.543
	**N**	5	2.9%	3	4.5%		
	**D and SD**	3	1.7%	0	0.0%		
**12. Most nursing courses can be offered through CBL method**	**A and SA**	169	98.3%	64	97.0%	1.651	0.501
	**N**	2	1.2%	2	3.0%		
	**D and SD**	1	0.6%	0	0.0%		
**13- Increases students’ self-confidence**	**A and SA**	166	96.5%	64	97.0%	0.535	1.00
	**N**	5	2.9%	2	3.0%		
	**D and SD**	1	0.6%	0	0.0%		
**14. Reduces class monotony**	**A and SA**	165	95.9%	65	98.5%	0.626	1.00
	**N**	5	2.9%	1	1.5%		
	**D and SD**	2	1.2%	0	0.0%		
**15. Makes students think deeply**	**A and SA**	165	95.9%	64	97.0%	1.309	0.559
	**N**	6	3.5%	1	1.5%		
	**D and SD**	1	0.6%	1	1.5%		
**16. There is possibility for more questions and answers**	**A and SA**	168	97.7%	65	98.5%	0.550	1.00
	**N**	3	1.7%	1	1.5%		
	**D and SD**	1	0.6%	0	0.0%		
**17. If this method is used in clinical practice, it will be more efficient**	**A and SA**	166	96.5%	65	98.5%	0.772	0.815
	**N**	3	1.7%	1	1.5%		
	**D and SD**	3	1.7%	0	0.0%		
**18. Students’ information is well assessed and evaluated**	**A and SA**	169	98.3%	65	98.5%	0.758	1.00
	**N**	2	1.2%	1	1.5%		
	**D and SD**	1	0.6%	0	0.0%		
**19. Summarizing the contents is easy**	**A and SA**	167	97.1%	64	97.0%	1.009	0.802
	**N**	4	2.3%	1	1.5%		
	**D and SD**	1	0.6%	1	1.5%		
**20. In general, CBL method is better than lecture-based learning**	**A and SA**	169	98.3%	63	95.5%	2.160	0.234
	**N**	2	1.2%	2	3.0%		
	**D and SD**	1	0.6%	1	1.5%		

*A and SA* Agree and strongly agree, *D and SD* Disagree and strongly disagree, *N* Neutral

Although female students constituted the majority of the participants (72.3%) compared to males (27.7%), no significant gender-based differences were observed in perception levels regarding the various features of CBL, as indicated by each question (*P* > *0.05*).

### Overall perception level with the incorporation of CBL into the traditional teaching framework applied physiology and its association with genders and age groups

Overall, 232 students (97.5%) affirmed that the combined CBL with TTMs approach is superior to TTMs alone (Table 3). Figure S3 illustrates the overall perception levels of enrolled students after integrating CBL with TTMs in teaching applied physiology during the neuroscience course for undergraduate physiotherapy students. The perception scores ranged from 25.0 to 100.0, with a median of 99.0 and an IQR of 88.0–100.0.

Table 4 indicates a significantly higher perception level among female participants compared to males (*P* < *0.05*), with a median perception score of 100.0 for females compared to 96.5 for males. Similarly, the mean rank of the total perception score was 124.65 for females and 106.08 for males. In contrast, no significant differences (*P* > *0.05*) were observed in total perception scores across different age groups. The median and mean rank values were 99.0 and 121.52, respectively, for the 18–20 age group, compared to 96.5 and 99.68 for the 21–23 age group.

**Table 4:** Comparison of the total perception score of the hybrid approach “integrated CBL with traditional teaching” among different genders and age groups

		**Total Perception score**				**Mann–Whitney U test**	
		**Median**	**25**^**th**^ **Percentile**	**75**^**th**^ **Percentile**	**Mean rank**	**Z**_**mw**_	***P*-Value**
**Gender**	**Female**	100.0	89.5	100.0	124.65	−1.966	0.049*
	**Male**	96.5	86.0	100.0	106.08		
**Age group**	**18–20**	99.0	88.0	100.0	121.52	−1.496	0.135
	**21–23**	96.5	85.0	100.0	99.68		

^*^Significant at *p* < 0.05

### Student’s academic achievement

Integrating CBL with TTMs in teaching applied physiology was apparently associated with better academic achievement. Although the maximum grades were the same between the two methods of teaching (10), the minimum grade was 2 out of 10 when applied physiology was taught through the TTMs in the first semester, compared to a minimum grade of 7.5 out of 10.0 with the integration of CBL with TTMs in the fourth semester.

Similarly, the median grade was 8.5 with TTMs alone compared to 10 when CBL was combined with traditional methods. The better achievement of the students was also apparent at both the 25^th^ and 75^th^ percentiles, where the hybrid approach integrating CBL with TTMs scored 10.0 and 10.0 at both the 25^th^ and 75^th^ percentiles, respectively, compared to 7.0 and 9.5 in TTMs (Table 5).

**Table 5:** Comparison of the students’ grades between traditional alone and the combined case-based with traditional teaching methods

Grades	Minimum	Maximum	Median	25^th^ Percentile	75^th^ Percentile	Z	*P*-Value
TM							
**Traditional**	2.0	10.0	8.5	7.0	9.5	−11.668	< 0.001*
**CBL**	7.5	10.0	10.0	10.0	10.0		

*CBL* case-based learning, *TM* teaching method

^*^Significant at *p* < *0.05*

### Facilitators’ feedback

CBL was well received by the facilitators, with 85% agreeing that combining CBL with TTMs enhances students’ communication skills, fosters a better and safer relationship between facilitators and students, and helps in understanding group dynamics. Additionally, 80% of the facilitators supported incorporating CBL into the timetable as a regular learning tool along with TTMs (Figure S4).

A substantial proportion (80%) of the facilitators believed that integrating CBL with TTMs is a better teaching strategy, as it enhances students’ problem-solving and self-directed learning abilities while facilitating the integration of knowledge across different subjects. Additionally, 80% of facilitators emphasized that this approach supports better knowledge retention among students (Figure S4). The majority also agreed that combining CBL with TTMs represents a concerted effort to bridge existing gaps between physiologists and students in a clinical context.

While most facilitators welcomed CBL as a complementary tool to TTMs, aiding in the achievement of objectives, some challenges were identified by the facilitators and are summarized in Table 6.

**Table 6:** Challenges faced by facilitators during CBL implementation in applied physiology

Challenge	Description
**Resistance to change**	Some students described CBL as challenging or unfamiliar and felt comfortable with existing traditional methods
**Student engagement**	Some students, especially those who are shy, may struggle to actively engage with the CBL approach, with reduced participation and learning outcomes. The motivation of non-participant students to effectively engage in the case discussion could be challenging
**Differential student preparedness**	Students may enter the CBL sessions with different levels of preparedness, which could potentially hinder the efficacy of group analysis and discussion
**Time restriction**	CBL can be time-consuming, particularly with group work, dynamics, discussions, and reflection. This element of time constraints could be challenging and limit the depth and scope of the learning experience
**Time management**	The delicate balance between the case studies wrapped up within the allocated time frame for the course can be challenging and potentially impact the depth of discussion and analysis
**Facilitators’ Training**	Adequate or persistent facilitators’ training is required to ensure their ability to effectively guide CBL, manage group dynamics, and provide constructive feedback

## Discussion

Despite the benefits of TTMs as a learning approach in undergraduate medical teaching, they have been questioned due to a lack of reasoning skills and critical thinking [26], which are key elements in any physiotherapy career. CBL offers a dynamic and interactive, learning strategy that integrates both guided and structured learning to cultivate these crucial abilities [27].

In the current study, CBL was concomitantly applied with TTMs in applied physiology to undergraduate physiotherapy students encourages the active students’ learning and yields a more productive outcome as an exploratory step that reflect the potential benefits of CBL in fostering engagement and comprehension. By discussing different real clinical case scenarios related to the topics taught in the neuroscience module, physiotherapy students evaluated their own perception level while their academic achievement was assessed through their grades.

The higher overall perception level observed with integration of CBL within TTMs in the current study underscores the importance of CBL in preparing students for the clinical demands of their medical careers while fostering essential skills critical for their performance and competency [28].

The analysis of student perceptions demonstrates strong support for integrating CBL with TTMs across all 20 measured items. Remarkably, 97.5% of students expressed a positive perception of the combined approach compared to TTMs alone, with an overall perception score of 99.0 out of 100.

Students particularly appreciated CBL’s ability to facilitate deeper thinking and its effectiveness in reducing class monotony. These findings suggest that CBL when integrated with TTMs may be particularly beneficial for maintaining student interest and motivation throughout the course.

The high level of student perception with CBL’s ability to organize information and facilitate assessment indicates that this method may also improve students’ learning outcomes. Furthermore, the perception that the combined learning approach increases students’ self-confidence aligns with previous research suggesting that active learning approaches can enhance student confidence [29]. Interestingly, students perceived the combination of CBL and TTMs as more efficient for clinical practice. This suggests that integrating CBL with TTMs may offer benefits beyond academic settings, potentially enhancing clinical decision-making skills [30].

The positive perceptions observed in this study can be attributed to the student-centered nature of CBL, which fosters active participation, critical thinking, and real-world applicability [31]. These features address many limitations of TTMs, such as passive learning and lack of engagement [32]. Additionally, the structured design of CBL cases, which align closely with course objectives, ensures clarity and focus in learning [33].

The high students’ perception with CBL in teaching physiology was reported previously, where students clearly enjoyed their experience with CBL, perceived it as valuable, and gave an overall rating of the CBL program as good—excellent on a five-point Likert Scale [34].

The current findings are consistent with the preliminary results reported by Brown et al. (2012) [35], who implemented a CBL approach for undergraduate health sciences students at the University of Ottawa. In their pilot project, 144 students participated and achieved an average score of 4.13 out of 5 on a quiz designed to evaluate their mastery of the concepts covered in the CBL sessions. Furthermore, the students rated the overall learning benefit of the program as 3.82 out of 4 on a nominal scale, highlighting its perceived educational value [35]. These results reinforce the potential of CBL to significantly enhance the learning experience for undergraduate students, especially when combined with TTMs.

The positive findings from the current study are further bolstered by the recent work of Saini et al. (2024), who highlighted CBL, as a student-centric, self-directed learning approach that fosters collaboration and critical thinking among 134 final-year physiotherapy, medical, and nursing students [36]. Using pre- and post-test assessments, they reported significantly higher post-test scores following the CBL approach, indicating higher knowledge acquisition (*p* < *0.05*) [36]. Students also reported enhanced learning experiences, highlighting the role of CBL in consolidating and integrating knowledge, and applying learned concepts to real-world scenarios.

To investigate potential gender-based differences in the effectiveness of CBL integration with TTMs, we assessed the correlation between gender and perceptions. Our findings showed no significant gender-based differences in perception levels regarding the various features of CBL (Table 4). These results are consistent with previous studies that also reported no significant gender differences in the perception of active learning methods [37]. In contrast, other educational research has highlighted the presence of gender-based differences in perceptions across broader educational contexts [38, 39].

CBL emphasized on the active role of the students in creating their own knowledge (discovery learning) in CBL while engaging with the designed clinical cases and building their own understanding of medical procedures and concepts [40]. It has been stated that CBL provides better opportunities for students to formulate diagnoses and delineate the appropriate management solutions as well as to relate the underlying possible mechanisms to the proposed diagnosis and treatment [41].

CBL was reported by students to enhance their motivation and interest in learning, based on their experiences during the course. Interestingly, CBL-related student-centered features, such as self-confidence, learning, and critical thinking, were all higher with CBL (Table 3). These findings support the idea that these skills could be improved through CBL.

Additionally, the implementation of CBL as a complement to TTMs in applied physiology was linked to more cooperation between students with better teacher-student relationship, creating trusting and collaborative classroom culture. Prior lines of evidence pointed out that CBL encouraged students to share their knowledge during discussion and resolve the clinical cases [42, 43]. Indeed, inter-peer collaboration and interaction are considered fundamental skills required to efficiently work in multidisciplinary clinical teams.

The displayed results in this study are consistent with the prior results that scored CBL as an effective learning tool in developing critical thinking skills, allowing students to link what they have learned with real-world scenarios [41, 44].

Combined CBL with TLMs application was associated with significantly promoted academic performance (Table 6). In alignment with our results, the application of CBL in teaching endocrine physiology was associated with enhanced students’ learning with better knowledge assimilation. The improved retention of knowledge with CBL could be attributed to the fact that students are required to study simultaneously the same topic from all subjects and integrate the knowledge to proceed to decision-making for the given problem in the case scenario [25].

## Facilitator Perspectives on CBL

The facilitators believed that integrating CBL to TTMs would better prepare physiotherapy students for future clinical practice by challenging them with realistic clinical cases. Additionally, they speculated that students are expected to collaboratively apply their previously acquired theoretical knowledge to gradually make appropriate decisions and propose solutions to the assigned clinical scenarios while identifying the key relevant characteristics. The facilitators exhibited a relatively low effort in providing detailed information about the clinical case while stimulating a gradual discussion among students.

The reported positive feedback by the facilitator in the current study matched that previously recorded in CBL implementation studies [2, 25].

The facilitators reported that students became more actively engaged and collaborative during the discussion of clinical cases in CBL sessions. This observation was supported by the thematic analysis of open-ended questions. The qualitative feedback highlighted that students were more motivated to participate, ask questions, and share their insights during the discussion of clinical cases. This could be explained by the way CBL implementation depends on open questions, which leaves the students more confident and promotes their participation in the clinical discussion [45, 46].

## Strengths points of the current study

This study brings several unique strengths to the field of physiotherapy education. It is the first to integrate CBL along with TTMs in applied physiology specifically for physiotherapy students, providing novel insights into the effectiveness of this combined approach in this context. The present study focuses on neurophysiology, a critical area in physiotherapy, enhancing students’ skills in managing clinical cases with a neurological basis.

Secondly, the careful formulation of course objectives and selection of real and previously published cases by a committee of physiologists and neurologists, in consultation with students, ensured that the CBL approach was tailored to the specific needs and interests of the target students.

Additionally, the relatively large sample size (*n* = 238) and high response rate (97.5%) of the student survey provide robust evidence for the effectiveness of integrating CBL with TTMs. The structured weekly implementation in lab sessions with OSPE-based assessment underscores a replicable and adaptable model, presenting a practical framework for other institutions aiming to enhance applied physiology education.

## Limitations of the current study

The current study has some limitations, including involvement of one cohort, convenience sampling and the single-institution setting, which may limit generalizability of the findings. Although this study primarily focused on short-term outcomes at the end of the semester, the strong academic performance observed provides a foundation for hypothesizing that the active learning environment created by CBL can support long-term knowledge retention. To address this limitation, future research could include longitudinal assessments, such as follow-up exams or evaluations of clinical performance to measure the durability of knowledge retention. Additionally, incorporating spaced repetition or periodic review sessions into the CBL framework may further enhance long-term retention. Moreover, it would be better if we practiced CBL over a longer period of time in a wider range of labs equipped with appropriate infrastructure and incorporating a newly developed online learning environment.

Finally, this study is limited by the content differences between the first and fourth semesters that might have influenced outcomes, potentially confounding the comparison of academic performance. Additionally, the absence of a control group may be considered another limitation. Future research should consider incorporating a control group to enable a more robust and direct comparison.

## Enhancing CBL implementation for optimizing practice in the future

While CBL holds immense potential to transform applied physiology education into a clinical context, its implementation could present unique challenges. These potential challenges of implementing CBL in applied physiology could be addressed through proposed strategies based on our experience; illustrated in Table 7. These suggestions could help to construct a better CBL framework and empower students to become active participants for better engagement in CBL. By addressing these challenges, students could acquire a broad scale of critical thinking abilities and collaboration skills vital to adequately preparing them for the complexities of their future clinical practice.

**Table 7:** Key considerations and proposed strategies for better CBL implementation in applied physiology in the future

CBL- related category	Themes	Description
**Training**	**Faculty members’ training**	Provide continuous training for staff members to effectively facilitate CBL sessions and provide better constructive feedback to students
	**Students training**	Students should be adequately trained on CBL techniques to attain maximal benefits from and fully realize the CBL
**Facilitated Learning**	**Learning resources**	Provide access to a wide range of different resources, such as research articles, textbooks, and online materials, to support students in understanding and analyzing the selected case scenarios
**Application of Knowledge**	**Interdisciplinary collaboration**	Encourage close collaboration with other healthcare disciplines to construct comprehensive case scenarios that mirror real-world healthcare scenarios
	**Technology integration**	Explore the use of technology- based educational tools to promote CBL experiences, such as interactive learning platforms and virtual simulations
**Case Construction and Presentation**	**Interdisciplinary integration**	Construction of integrating case scenarios that reflect interdisciplinary healthcare scenarios with better collaboration between different healthcare disciplines
	**Interactive presentations**	Interactive cases’ presentations enhance deeper understanding and encourage active students’ participation by enabling students to present selected cases live, such as through simulation
**Group Discussion**	**Group discussion guidelines**	Establish clear guidelines to facilitate group discussions in order to ensure effective collaboration among students and more broad knowledge sharing
**Reflection**	**Reflection and feedback mechanisms**	Implement mechanisms for students to reflect on their CBL experiences and provide feedback on the effectiveness of CBL in applied physiology
**Students’ Assessment**	**Effective students’ evaluation**	Developing new, effective approaches to students’ evaluation rather than traditional methods of grading/ testing that better fit with the CBL application
	**Assessment rubrics**	Establishing assessment rubrics to accurately evaluate students’ understanding of applied physiology concepts and their application within the context of CBL
**Long-Term Impact**	**Continuous improvement and evaluation**	Regular evaluation of the effectiveness of CBL and making mandatory adjustments to enhance the learning experience Develop a plan for continuous improvement based on feedback and assessment data to refine the CBL implementation
	**Follow long-term impact**	Collecting extensive long-term data over an extended time frame could be crucial to gauge the long-term impact of CBL

## Future research directions

CBL can be broadly implemented as a more interactive teaching tool not only in applied physiology but also in other health sciences to overcome the limitations of TTMs and ensure better outcomes.

While the current study focuses on short-term academic performance as an indicator of concurrent TTMs and CBL’s effectiveness, we recommend implementing follow-up assessments in subsequent semesters or at the end of the program to capture the long-term impact of CBL on knowledge retention. These assessments should test foundational concepts gained during physiology courses to evaluate the longevity of knowledge retention. Additionally, future studies could explore the perception and effectiveness of CBL when implemented independently of TTMs, enabling a clearer understanding of its isolated impact on student learning outcomes.

Based on the positive results of the current study, we recommend integrating CBL in other courses within the physiotherapy program. This consistent application of active learning strategies can reinforce knowledge retention through more engagement and motivation of students, leading to better encoding and retrieval of information and promoting better academic outcomes. Additionally a longitudinal study should be conducted to track student performance and knowledge retention throughout the physiotherapy program that would provide valuable insights into the long-term effects of CBL. Also these studies would be instrumental in assessing clinical competence and patient outcomes.

## Conclusion

Incorporation of CBL into the existing TTMs framework for teaching applied physiology was advantageous for physiotherapy students as a preliminary step for their entry into clinical practice and ultimately in successfully managing patients, as it encourages students to pursue self-directed learning and to develop both analytical as well as problem-solving skills. This hybrid teaching tool with integration of CBL in applied physiology encourages active learning, helps physiotherapy students gain the requisite knowledge, and enhances their analytical and communication skills.

The interactive and contextually relevant nature of CBL accommodates different learning styles, catering to visual, auditory, and kinesthetic learners alike. By engaging students in real-world scenarios, CBL fosters critical thinking, problem-solving, and clinical reasoning skills, all of which are essential for professional practice.

## Supplementary Information

Supplemenentary Material 1.

## Data Availability

All the generated or analyzed datasets during the current study are readily available from the corresponding author on any reasonable request. The authors declare no competing interests.

## Abbreviations

CBL: Case-based learning
UPS: Undergraduate physiotherapy students
SD: Socio-demographic
SPSS: Statistical Package for the Social Sciences
IBM: International Business Machines Corporation
CVI: Content Validity Index
CVR: Content Validity Ratio
BSS: Brown-Séquard syndrome
HS: Horner’s syndrome
DN: Diabetic polyneuropathy
AC: Ankle clonus
