# Supplementary material for: Bringing theory to life: integrating case-based learning in applied physiology for undergraduate physiotherapy education
Source: BMC Med Educ. 2025 Feb 13;25:234. doi: 10.1186/s12909-025-06725-7 (PMC11827333; doi:10.1186/s12909-025-06725-7)
Supplement: Supplementary file 1 — Supplemenentary Material 1. [file 12909_2025_6725_MOESM1_ESM.docx]

**Bringing Theory to Life: Integrating Case-Based Learning in Applied Physiology for Undergraduate Physiotherapy Education**


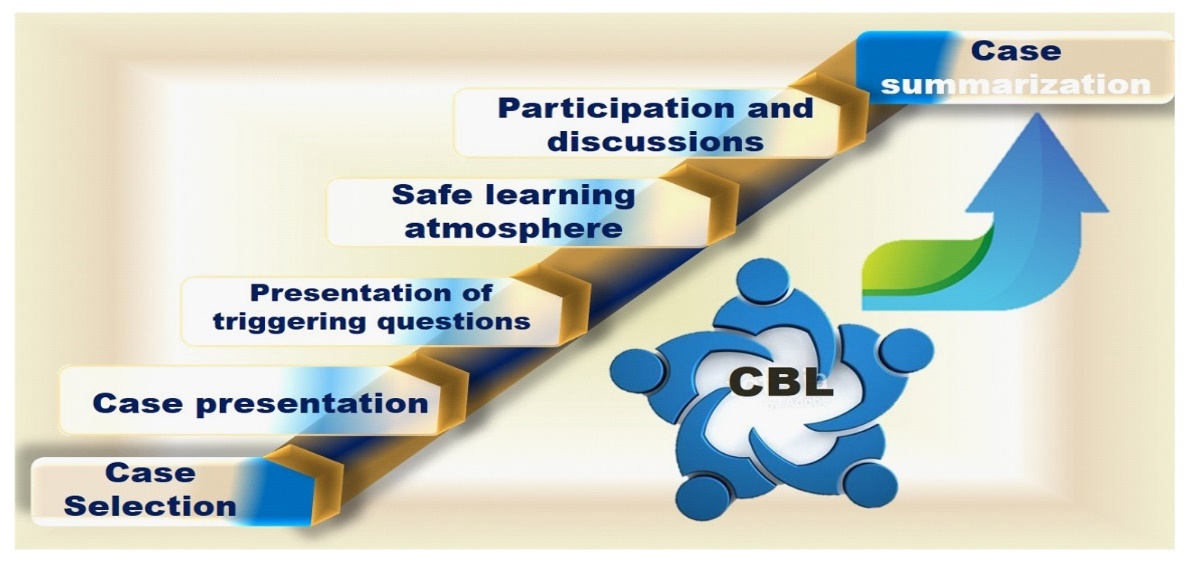


**FIGURE S1. Framework for implementing case- based learning in applied physiology**

**FIGURE S2. Distribution (percentages) of gender and age groups among the study participants**


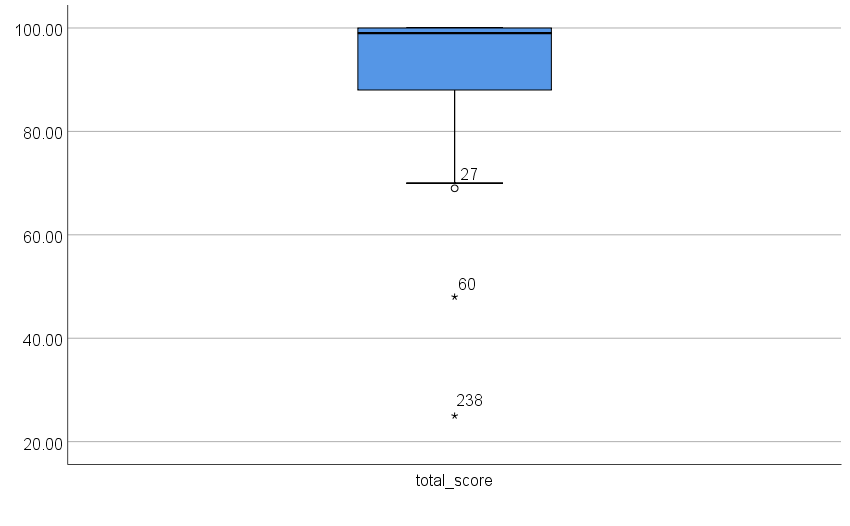


**FIGURE S3. Boxplot of overall perception score of combining CBL with traditional teaching in applied physiology**

**FIGURE S4. Facilitators response according to a five-point Likert scale.**

**Q1**: CBL is a better method of teaching/learning than the conventional one

**Q2**: CBL promotes self-study and problem-solving abilities of the students.

**Q3**: CBL helps in the recall and application of basic sciences to the given clinical scenario.

**Q4**: CBL helps in better retention of knowledge.

**Q5**: CBL helps in improving communication skills of the students

**Q6**: CBL helps in understanding the principles of group dynamics

**Q7**: CBL deprives students of an opportunity to acquire knowledge from experienced and good teachers

**Q8**: CBL gives opportunity for even staff members with poor teaching skills to be good facilitators

**Q9**: CBL facilitates a better and healthy teacher-student relationship

**Table 1S. Face validity index (FVI) by 20 physiotherapy undergraduate students.**

| **Item No. in**  **P Questionnaire** | **PS1** | **PS2** | **PS3** | **PS4** | **PS5** | **PS6** | **PS7** | **PS8** | **PS9** | **PS10** | **PS11** | **PS12** | **PS13** | **PS14** | **PS15** | **PS16** | **PS17** | **PS18** | **PS19** | **PS20** | **FVI^a^** |
| --- | --- | --- | --- | --- | --- | --- | --- | --- | --- | --- | --- | --- | --- | --- | --- | --- | --- | --- | --- | --- | --- |
| 1 | 4 | 4 | 4 | 4 | 4 | 3 | 4 | 4 | 3 | 2 | 3 | 3 | 4 | 4 | 4 | 4 | 4 | 4 | 4 | 4 | 0.95 |
| 2 | 4 | 3 | 3 | 4 | 3 | 2 | 4 | 4 | 3 | 4 | 3 | 2 | 4 | 4 | 3 | 4 | 4 | 4 | 3 | 4 | 0.9 |
| 3 | 4 | 4 | 4 | 4 | 4 | 4 | 4 | 4 | 3 | 4 | 4 | 3 | 4 | 4 | 4 | 4 | 4 | 4 | 4 | 4 | 1.0 |
| 4 | 3 | 3 | 4 | 4 | 4 | 4 | 4 | 4 | 4 | 2 | 4 | 4 | 3 | 4 | 4 | 4 | 4 | 3 | 4 | 4 | 0.95 |
| 5 | 4 | 3 | 4 | 4 | 4 | 3 | 3 | 4 | 4 | 4 | 3 | 2 | 4 | 4 | 4 | 4 | 4 | 4 | 3 | 4 | 0.95 |
| 6 | 2 | 3 | 4 | 4 | 4 | 3 | 4 | 3 | 4 | 4 | 3 | 4 | 4 | 4 | 4 | 4 | 4 | 3 | 2 | 3 | 0.9 |
| 7 | 3 | 4 | 4 | 4 | 4 | 4 | 4 | 3 | 2 | 4 | 3 | 3 | 4 | 4 | 4 | 2 | 2 | 4 | 4 | 4 | 0.85 |
| 8 | 3 | 4 | 4 | 4 | 4 | 4 | 4 | 3 | 4 | 3 | 4 | 4 | 4 | 4 | 4 | 4 | 4 | 4 | 4 | 4 | 1.0 |
| 9 | 3 | 4 | 4 | 4 | 4 | 4 | 4 | 4 | 4 | 4 | 4 | 4 | 4 | 4 | 4 | 4 | 4 | 4 | 3 | 4 | 1.0 |
| 10 | 2 | 4 | 4 | 4 | 4 | 4 | 3 | 4 | 4 | 4 | 3 | 4 | 4 | 4 | 4 | 4 | 4 | 4 | 4 | 4 | 0.95 |
| 11 | 4 | 4 | 4 | 4 | 4 | 2 | 4 | 3 | 4 | 4 | 4 | 2 | 4 | 4 | 4 | 4 | 4 | 3 | 3 | 4 | 0.9 |
| 12 | 4 | 4 | 4 | 4 | 4 | 3 | 4 | 3 | 4 | 4 | 4 | 4 | 4 | 4 | 4 | 4 | 4 | 4 | 4 | 4 | 1.0 |
| 13 | 4 | 3 | 2 | 4 | 3 | 3 | 4 | 4 | 4 | 2 | 2 | 4 | 4 | 4 | 3 | 4 | 4 | 4 | 3 | 4 | 0.85 |
| 14 | 4 | 3 | 4 | 4 | 4 | 3 | 3 | 4 | 3 | 4 | 4 | 4 | 4 | 3 | 3 | 4 | 4 | 4 | 4 | 4 | 1.0 |
| 15 | 3 | 4 | 4 | 4 | 4 | 4 | 4 | 4 | 4 | 4 | 4 | 4 | 4 | 4 | 2 | 4 | 4 | 4 | 4 | 4 | 0.95 |
| 16 | 4 | 3 | 3 | 4 | 4 | 4 | 4 | 4 | 4 | 4 | 3 | 4 | 4 | 4 | 3 | 4 | 4 | 4 | 3 | 4 | 1.0 |
| 17 | 4 | 2 | 3 | 4 | 4 | 4 | 4 | 4 | 4 | 4 | 4 | 4 | 4 | 4 | 4 | 4 | 4 | 4 | 3 | 4 | 0.95 |
| 18 | 2 | 4 | 4 | 2 | 4 | 4 | 4 | 4 | 4 | 4 | 4 | 4 | 4 | 4 | 4 | 3 | 4 | 4 | 4 | 4 | 0.9 |
| 19 | 3 | 3 | 4 | 4 | 4 | 4 | 4 | 4 | 4 | 4 | 4 | 4 | 4 | 4 | 4 | 4 | 4 | 4 | 4 | 4 | 1.0 |
| 20 | 4 | 4 | 4 | 4 | 4 | 4 | 4 | 4 | 4 | 4 | 4 | 3 | 4 | 3 | 4 | 4 | 4 | 4 | 4 |  | 1.0 |

^a^FVI = Face Validity Index. The average value of the face validity index was 0.95
